# Governance factors in the identification of global conservation priorities for mammals

**DOI:** 10.1098/rstb.2011.0114

**Published:** 2011-09-27

**Authors:** Johanna Eklund, Anni Arponen, Piero Visconti, Mar Cabeza

**Affiliations:** 1 Metapopulation Research Group, Department of Biosciences, University of Helsinki, PO Box 65, 00014 Helsinki, Finland; 2 Australian Research Council Centre of Excellence for Coral Reef Studies, James Cook University, Townsville, Queensland, Australia; 3 Global Mammal Assessment programme, Department of Biology and Biotechnologies, Sapienza Università di Roma, Viale dell'Università 32, 00185 Rome, Italy; 4 Biodiversity and Global Change Lab, Museo Nacional de Ciencias Naturales, CSIC, C/José Gutiérrez Abascal 2, 28006 Madrid, Spain

**Keywords:** spatial conservation prioritization, mammals, good governance, corruption, conservation effectiveness

## Abstract

Global conservation priorities have often been identified based on the combination of species richness and threat information. With the development of the field of systematic conservation planning, more attention has been given to conservation costs. This leads to prioritizing developing countries, where costs are generally low and biodiversity is high. But many of these countries have poor governance, which may result in ineffective conservation or in larger costs than initially expected. We explore how the consideration of governance affects the selection of global conservation priorities for the world's mammals in a complementarity-based conservation prioritization. We use data on Control of Corruption (Worldwide Governance Indicators project) as an indicator of governance effectiveness, and gross domestic product *per capita* as an indicator of cost. We show that, while core areas with high levels of endemism are always selected as important regardless of governance and cost values, there are clear regional differences in selected sites when biodiversity, cost or governance are taken into account separately. Overall, the analysis supports the concentration of conservation efforts in most of the regions generally considered of high priority, but stresses the need for different conservation approaches in different continents owing to spatial patterns of governance and economic development.

## 1. Introduction

Nearly one quarter of mammal species are globally threatened or already extinct [1]. Despite major conservation efforts, mammals continue to decline, and at faster rates than birds, with threats such as habitat loss and hunting being still ineffectively addressed [2]. During the last 500 years, 76 mammal species have been lost, and the rate is accelerating [1]. These numbers call for additional conservation efforts and careful identification of priorities.

Global conservation priorities have typically been identified based on information on species richness, endemism and areas under most threat [3–5]. These ‘hotspot’ priorities have been criticized by the rising field of systematic conservation planning [6], which addresses resource allocation problems with quantitative objectives through optimization techniques. Such an approach is customizable to include a number of relevant factors. Mammals, in particular, have been the target of several applications of this type, accounting for conservation costs [7], human pressure [8], latent extinction risk [9] and opportunity costs to limit conflicts between conservation and agricultural activity [10].

The field of systematic conservation planning has brought along a strong focus on the need to account for economic costs in the conservation planning process. This is necessary in a world of limited resources, but comes with its own problems (see [11] for a more comprehensive discussion on the topic). At global scales, when costs are considered, developing countries tend to be prioritized as they offer high biological diversity with potentially low land acquisition and management costs [12]. However, socio-political differences between countries are arguably no smaller or less important than the economical ones. It has indeed been shown that conservation outcomes correlate with socio-political factors [13,14], with low values for socio-political governance indicators corresponding to poor conservation outcomes. For example, the numbers of African Elephants and Black Rhinos could be explained by corresponding national values of a corruption indicator [15] and additional correlative relationships supporting this fact have been found for the Protected Area Management Effectiveness Index and the Human Development Index [16], and tropical protected area effectiveness and corruption levels [17].

Many countries with high species richness and endemism could offer low costs for conservation, but also suffer from inefficient governance, political instability and higher levels of corruption [13]. Thus, while the benefit-to-cost ratio of conservation may be apparently greater in developing regions of the world because of their lower costs for land acquisition and management, it is often exactly in these countries that the lack of good governance impeding effective conservation is most acute.

To date, governance has rarely been accounted for in conservation planning approaches (but see [14] and [18]). However, governance should not be disregarded, even though we lack decent models on how corruption impacts on conservation success and the relationship between the two still is rather unexplored [19,20]. The problem is the lack of empirical data to explore this relationship which could then be used in quantitative prioritizations either by penalizing the effectiveness of conservation actions or by increasing the costs for carrying them out. Despite the lack of quantitative measures to account for this socio-political dimension, many still acknowledge the importance of it [8,10,21,22].

The aim of this study is to compare mammal conservation priorities in relation to governance and economic indicators. The availability of recently completed habitat suitability models for terrestrial mammals [23] allows for the exploration of this on a global scale but with fine resolution data. The global focus allows us to explore the full variation in costs for conservation and governance. We applied a systematic conservation planning approach aimed at prioritizing important mammal conservation regions, while accounting for mammal diversity only, mammal diversity conditional to costs, mammal diversity penalized by governance and various combinations of these. Here, we assume that corruption has a negative impact on conservation effectiveness, but see §4 for alternative hypotheses. Our study allows the exploration of trade-offs between these factors and it also generates balanced solutions that consider biodiversity together with economical costs and quality of governance. But first and foremost, it contributes to the debate on the importance of considering governance issues when aiming at effective conservation planning.

## 2. Material and methods

### (a) Mammal species distribution data

Distribution data for the world's terrestrial mammals come from habitat suitability models [23]. This high resolution dataset contains the modelled distributions of 5086 mammal species, and is based on the global mammal assessment led by the International Union for Conservation of Nature [1]. The data were used at a resolution of 0.1° and this resulted in 3600 × 1800 grid cells.

### (b) Cost data

Unlike Bode *et al*. [22] and Wilson *et al*. [18], we chose not to use the cost model derived in Balmford *et al*. [12] to estimate conservation costs at national level, because it reflects differences in the types of conservation projects that have been so far funded in different nations—wealthy nations engaging in more costly conservation actions (restoration, etc.) than the poor ones. Indeed, it results in a variation of seven orders of magnitude in costs between countries. Instead, as in other prioritization approaches [24], we use a more general indicator of cost, gross domestic product (GDP) *per capita*, which has a variation of *ca* three orders of magnitude. We used the nominal GDP *per capita* instead of GDP at Purchasing Power Parity, because global conservation prioritizations are likely to be relevant for international non-governmental organizations or donor investments from industrialized countries [25], and thus investments must be made using market exchange rates. Economic and population data were obtained from the World Development Report 2009 [26], and used to calculate GDP (nominal) *per capita*. This information was available for 182 countries.

### (c) Governance data

The most comprehensive and publicly available measure of governance is the Worldwide Governance Indicators (WGI) project [27], which incorporates many of the other available assessments on governance (International Country Risk Guide, Freedom House, Country Policy and Institutional Assessment, and most of the sources also used by Transparency International in forming their composite indicator). These data are available for a larger set of countries and territories than individual indexes used previously in conservation studies (e.g. [15]). The WGI-dataset includes data on six dimensions of governance (voice and accountability, political stability and absence of violence/terrorism, government effectiveness, regulatory quality, rule of law and control of corruption) for 212 countries and territories over the period 1996–2007. These aggregated indicators are based on several hundred individual variables quantifying perceptions of governance, drawn from separate data sources constructed by different non-governmental organizations, private–public agencies and individuals (such as Afrobarometro, World Economic Forum, Gallup World Poll and Reporters without Borders). The aggregated indicators are weighted averages of the underlying data. The dataset has been used in some conservation studies [24,28], showing a strong positive correlation between national governance quality scores and GDP *per capita*, implying that many developing countries suffer from poor governance. All six governance factors are strongly correlated [24,28]. We chose to use a single factor, control of corruption, as corruption has been shown to be related to conservation outcomes [15] and also to influence the effectiveness of development aid [29].

### (d) Prioritization with Zonation

Global mammal priorities were identified with the conservation planning software Zonation [30,31]. The runs were performed with Zonation 3 development version, which is to be publicly released in 2011. Zonation produces a hierarchical prioritization of the landscape based on the biological value of sites, accounting for complementarity. The algorithm proceeds by removing the least valuable cell from the landscape, minimizing marginal loss of conservation value.

We used a variant of Zonation (Basic core-area Zonation) that encourages the representation of all species. The local biodiversity value of a cell is based on the species that has the highest proportion of its distribution remaining in the specific cell. In other words, the algorithm removes first cells with species that have wide distributions, and aims at retaining equal amounts of habitat for all species. When a cost layer (in this study, consisting of GDP and/or corruption scores—see below) is used, cell removal is based on local biodiversity value divided by cell cost. The Core-Area Zonation resembles the ‘gain metric’ of Wilson *et al*. [18] in that a species' distribution in a cell is valued relative to its total distribution, but with two main differences: (i) as cells are progressively removed, the importance of the occurrences of initially widely distributed species increases, and (ii) it produces a continuous ranking of the whole landscape, instead of using fixed targets for species representation or constraints for resource use.

Countries lacking the socio-political and economic data were masked out of the analysis, resulting in 179 countries used in our analyses (see electronic supplementary material, table S1 for the list of countries). Most of the excluded countries were small and unlikely to influence the general patterns in the results, but the absence of countries such as Afghanistan, Iraq and Somalia should be noted when interpreting the results. Species endemic to these countries were thus not included in the analysis, which meant having 5016 species in our results instead of the 5086 in the full dataset.

Data are not available for linking governance scores to effectiveness of mammal conservation, or to realized costs via empirically derived functions. In the absence of such models, we chose to incorporate governance scores in our objective function as increased costs, based on the assumption that in countries where the level of corruption is high only part of the resources will end up being used for the intended purpose. We explored how relative differences in cost and governance variables influence global conservation priorities. We re-scaled both variables to vary between 0 and 100. Eight different conservation scenarios were produced, one by letting only the biodiversity data drive the selection (referred to as the Biodiversity-only scenario, technically corresponding to cost layer values being equal everywhere), and seven scenarios by weighting and combining differently GDP and corruption data into a cost layer. We consider two extreme scenarios (Economic cost scenario and Governance scenario) by giving the maximum weight of 1 to one of the variables, and the minimum weight of 0 to the other. The equal weighting of economic costs and governance was also considered (giving 0.5 weights to both) and various weightings between these values (0.1, 0.25, 0.75, 0.9), always summing up to one (e.g. weight of 0.1 for governance, weight of 0.9 for economic cost). A final cost index was calculated as a combination of the two by a simple additive approach:
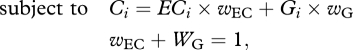
where *C*_*i*_ is the total cost considered for the grid cell *i*, *EC*_*i*_ is the economic cost class of cell *i* (based on GDP *per capita*), *G*_*i*_ is the governance cost class, and *w*_EC_ and *w*_G_ are weights given to the economic cost or the governance classes, respectively, as explained above. These different weighting schemes gave each country different cost-corruption indexes that were used as cost layers in the Zonation runs.

## 3. Results

**Figure 1.**
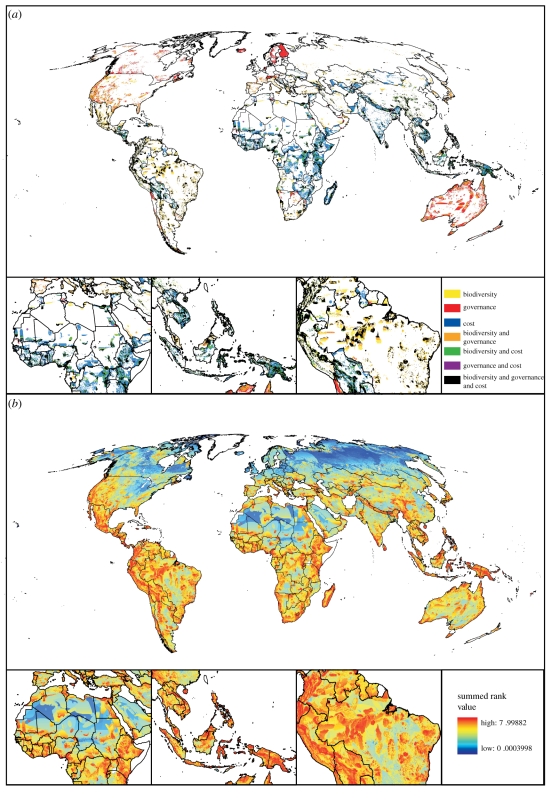
Spatial distribution of conservation priority areas selected in a complementarity analysis with different scenarios. (*a*) Top 10% priorities from the three most extreme scenarios: biodiversity only (yellow), costs only (blue) and governance only (red). Areas of overlap, between combinations of the scenarios are also shown: biodiversity + governance (orange); biodiversity and GDP (green); GDP and governance (purple). Areas identified as top 10% priorities across all three scenarios are shown in black. (*b*) Sum of ranks across all eight scenarios. Dark red indicates higher priorities across all scenarios, dark blue low priority across all scenarios. Intermediate colours indicate either intermediate importance or variable importance.

A comparison of the most extreme scenarios (Biodiversity-only, Economic cost and Governance scenarios) showed important continental differences in priorities (figure 1*a* and table 1; see also electronic supplementary material, table S1 for detailed results): Africa included many areas that stood out as priorities when economic costs were considered, but not when only biodiversity was accounted for (in blue). In South America, several priorities represented important areas for biodiversity, but higher costs combined with poor governance prevented them from being prioritized in the other scenarios (yellow). But variation in both costs and governance were large in South America, which showed in the priority map as higher variation in the colour patterns. Regions in the Northern Hemisphere and Australia stood out as important biodiversity areas with good governance (orange), but high costs prevented these regions from being selected when GDP *per capita* was considered. With highest penalties to poor governance some well-governed but expensive and partly less biodiverse areas e.g. in Australia and the Boreal region gained more emphasis (red).

**Table 1.** Five countries with largest areas in the top 10% fraction with the different prioritization scenarios. Area is given in thousands of square kilometres. The overlap column gives the area that overlaps between the three extreme solutions (governance, cost, biodiversity), which corresponds to the black areas in figure 1*a*.

	biodiversity		cost		cost 0.9		cost 0.75		cost 0.5		cost 0.25		cost 0.1		governance		overlap	
	Brazil	1504	China	893	Brazil	1452	Brazil	1533	Brazil	1468	Australia	1840	Australia	2207	Australia	2387	Brazil	658
	US	1034	Indonesia	888	China	1044	China	958	Australia	1268	Brazil	1288	US	1392	US	1401	China	515
	Australia	946	India	855	Indonesia	799	Indonesia	731	US	993	US	1276	Brazil	1157	Brazil	989	Indonesia	484
	China	936	Congo, DR	844	Mexico	656	Australia	716	China	826	China	705	China	631	Canada	683	Mexico	312
	Indonesia	699	Brazil	669	India	578	Mexico	688	Indonesia	661	Indonesia	595	Canada	579	China	551	Peru	288

If we focus on the top 10 per cent priorities in each scenario, the area that overlapped in all eight scenarios is small, covering only 5.68 per cent of the total land area. Common priorities, characterized by countries or regions with high endemism, were particularly common in Africa (overlap between the three extreme scenarios is shown in black in figure 1*a*). Coincidence was lowest between prioritizations done with the Governance versus the Economic cost scenario (black + purple in figure 1*a*), as would be expected given the correlation between GDP *per capita* and Control of Corruption (Kendall's tau 0.557, *p* < 0.001). Particularly, parts of Africa, and Southeast Asia showed larger proportions of low cost areas important for biodiversity but which are poorly governed (green in figure 1*a*).

When looking further than the top 10 per cent fraction, across all scenarios, a larger number of nations became important. figure 1*b* shows summed ranks across all scenarios, illustrating areas that were important with higher certainty no matter how costs and governance were considered (in red), and areas that were least valuable (in dark blue). Note that areas with intermediate summed rank values could either denote regions more sensitive to assumptions about influence of governance or cost (i.e. more variation between the different scenarios), or areas of consistently intermediate priority.

Correlation of cell ranks between different solutions were in accordance with the results in figure 1*a* (electronic supplementary material, table S2). They were very high between the closest scenario variants (e.g. the 0.1 weighting versus Governance scenarios is 0.994, *p* < 1^−15^) and lowest between the Economic cost and Governance scenarios (0.587, *p* < 1^−15^).

**Figure 2.**
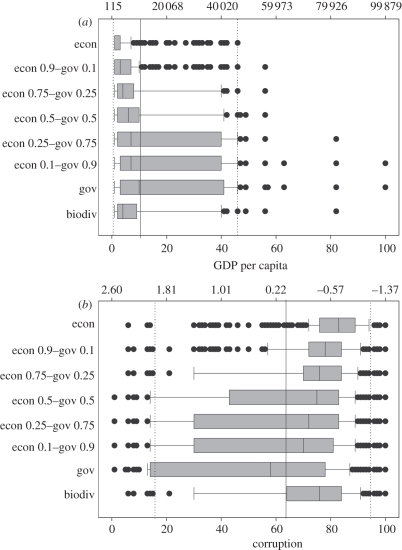
Distribution of GDP values (*a*) and governance scores (*b*) for all cells in top 10% fractions achieved with each of the eight scenarios. Mean and 95th percentiles across all nations in the dataset are shown as reference (solid line and dashed line, respectively). The units on *x*-axes are given both as the scaled values used in our calculations (below) and as the original GDP and Control of Corruption indicator values (above the panels).

Each scenario aimed at maximizing a particular objective, which included only biodiversity, or a combination of biodiversity and cost–governance constraints. The strongest penalties for governance resulted in more costly solutions, measured as the mean GDP *per capita* of selected units, but in the scenarios with intermediate weightings for cost and governance the overall cost of the solution was low (figure 2*a*), and similar to the cost of the solution when using biodiversity only. Solutions based on scenarios where GDP *per capita* has a high contribution resulted in the largest overall corruption level within the top priorities. These levels of corruption were much higher than the average corruption level across all nations (figure 2*b*). Those scenarios accounting for governance in the planning achieved substantial reductions in overall corruption levels. In turn, the intermediate Economic cost–Governance scenarios (0.5 weighting) had a cost distribution similar to the biodiversity-only solution, and provided an improvement in corruption levels compared with the biodiversity-only solution without incurring much increase in GDP *per capita* values.

**Figure 3.**
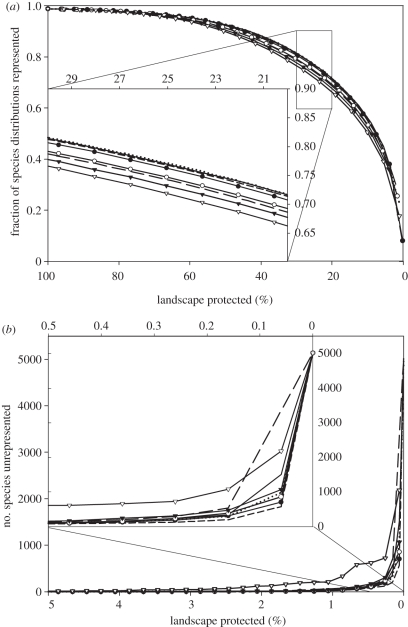
Species representation in all eight scenarios. (*a*) Mean proportion of species' distributions remaining protected at decreasing fractions of area protected. (*b*) number of species entirely unprotected at decreasing fractions of area protected. Note that the insets show a magnification corresponding to a particular range of percentage of landscape protected, between 20–30% for (*a*) and 0–0.5% in (*b*) (see the range of values on top of the inset). Dashed line, biodiversity only; long dashed line, governance only; solid line, economic 0.1–governance 0.9; dotted line, economic 0.25–governance 0.75; solid line with filled circles, economic 0.5–governance 0.5; solid line with open circles, economic 0.75–governance 0.25; solid line with filled inverted triangles, economic 0.9–governance 0.1; solid line with open inverted triangles, economic cost only.

There were small differences in the protection levels of species across scenarios (figure 3*a,b*). In terms of average proportion of the distribution protected, the GDP *per capita*-constrained solution achieved smallest overall coverage of species distributions, while the most extreme governance penalty resulted in intermediate levels. The intermediate Economic cost–Governance scenarios performed very similarly to the biodiversity-only solution. Nonetheless, all solutions achieved coverage of all species when 5 per cent or more of the world' terrestrial surface was protected (figure 3*b*). The Biodiversity-only scenario retained species furthest, as the fraction of protected area decreased, with most other scenarios achieving very similar results. Only the GDP *per capita*-constrained solution differed notably from other schemes, starting to lose species coverage at larger fractions of area protected (figure 3*b*).

## 4. Discussion

We have shown that there are important trade-offs between governance and cost, and that taking governance into account in the planning of conservation priorities, albeit simplistically, can make a large difference. Interestingly, we have shown that these trade-offs vary in space, some continents being more affected than others. Despite this, some regions are identified as priorities with any of the scenarios and thus we urge conservation efforts in these areas. We do not recommend neglecting poorly governed nations, and want to stress that different conservation approaches are needed in different regions.

Our intermediate Economic cost–Governance scenarios seem to provide desirable levels of governance with only a minor increase in cost, and are thus solutions that should be preferred. It is noteworthy that much more of the area important for biodiversity overlaps with the governance-based priorities than with the cost-based (more orange than green in figure 1*a*). Instead, cost-based priorities include much area that would not be prioritized in any other scenario. This illustrates the risks of focusing largely on economic costs [11], with funds being diverted from the most important biodiversity regions to areas where they might end up in the wrong hands, or anyhow used ineffectively. Incorporating costs into the prioritization process can be misleading if other socio-political constraints are not considered as well.

Important areas identified by other mammal conservation prioritization approaches also maintain high priority in our analysis (e.g. [8,10], see also similarities with Wilson *et al*. [18]). Our highest priority regions contain areas that despite poor governance are necessary to achieve global mammal conservation because of their high levels of endemism, e.g. the Andes, Mexico, Western Ghats, Madagascar, Papua New Guinea, Indonesia, Philippines, Sri Lanka, etc. Hotspot regions such as Japan, California and the Mediterranean basin are high priorities for biodiversity and also well-governed, but high costs would prevent them from being prioritized in a cost-based analysis. The top 10 per cent of the priorities identified by the Biodiversity-only, Economic cost and Governance scenarios overlapped by 40, 42 and 32 per cent, respectively, with the Biodiversity Hotspots [4].

We chose to incorporate governance in our prioritization as an additional cost. This is based on the perception that the apparent low costs of conservation in corrupt countries are not real: only a fraction of the invested conservation funds will be spent effectively on the intended purpose. Thus, to achieve the proposed goals, additional investments should be made, resulting in increased conservation costs. This is likely to be true, but not the whole truth. In poorly governed countries, even the conservation actions that end up being implemented may not be effective owing to poaching and other illegal activities, and corruption can act as an incentive for overexploitation of resources. So no matter how much economic resource is spent, the outcomes may remain unsatisfactory. An alternative (but technically largely equivalent) conservation prioritization approach would instead penalize the conservation outcomes based on quality of governance (i.e. assume that only a fraction of the biodiversity features can be protected effectively if the level of corruption is high). Wilson *et al*. [18] used a factor for ‘investment success’ derived from data on government ineffectiveness. But no matter which approach is chosen, empirical data to link effectiveness to such indicators (added cost, or effective conservation outcomes) are still required to achieve more reliable results.

On the other hand, our results showed dramatic impact of corruption penalties to spatial priorities, contrasting with the findings of Wilson *et al*. [18]. These differences can arise for at least two reasons: (i) a larger variation in cost data used by Wilson *et al*. [18] resulted in stronger influence of cost with respect to other factors, and (ii) the temporal variability in investment effectiveness considered by Wilson *et al*.'s dynamic approach, where investments made in periods of government ineffectiveness were considered to fail, and others to succeed despite following periods of ineffectiveness.

Corruption, and its influence on effectiveness of conservation programmes, has been identified as one of the ‘One hundred questions of importance to the conservation of global biological diversity’ [32]. If the assumptions about the positive relationship between good governance and conservation effectiveness hold, low cost solutions will not necessarily lead to effective use of resources: many of the countries that offer low cost investments for conservation actions are also the ones that suffer most from corruption. Yet, little effort has gone to date into considering governance in evaluating global conservation priorities.

A better understanding of the relationship between governance and conservation outputs, and governance and effective conservation costs will eventually allow for a better parametrization of the conservation approach presented here. Nevertheless, in the meantime lessons can perhaps be learnt from the field of aid effectiveness, where the discussion on how to account for governance factors has received major attention in the recent past. Studies have shown that aid will only lead to development (which should be considered the main aim of development assistance) in better governed countries [33–35]. That is, aid effectiveness seems to be conditional on the quality of institutions and policies. Better governed countries can use aid money more effectively, and therefore achieve expected development outcomes. These findings, still debated [36,37], have led to questions of selectivity versus conditionality [38,39]: should aid be given based on certain policy conditions (*ex ante*), or should it only be given to where it is likely to succeed (*ex post*)? These ideas may not apply directly to conservation, but there are clear analogies: should conservation investments be directed to regions with favourable conservation conditions, or should biodiversity needs drive conservation priorities but effectiveness be enhanced in problematic regions in different ways?

Corruption can have complex, both positive and negative, effects on biodiversity and these effects are likely to be different at different scales. Possible positive effects could be that a corrupt government is less likely to invest in infrastructure development in rural areas that would affect biodiversity negatively [20]. Corrupt countries experience inefficient economies and are less likely to attract foreign direct investments by multi-national corporations and industries to exploit their natural resources [20]. But wealthy nations do tend to displace the exploitative industries to poor countries, which might mean that the poor but less corrupt countries could face higher threats from industrial activities. On the other hand, slow economic growth is likely to affect local peoples' livelihoods negatively and this might again increase overexploitation on a local scale. We have ignored the potential positive effects of poor governance in this study, but our conservation planning framework could account both for positive and negative effects, once the relationship between governance and conservation effectiveness is better understood.

To conclude, we do not suggest that allocation of conservation resources should follow the priority maps produced in our analyses, although they may give some indication of regions that may deserve further considerations before large conservation investments are made. We warn about the use of economic costs in conservation prioritization exercises, when this is detached from other socio-economic factors, as this may result in resources being poured into either regions less important for biodiversity or ineffective projects. Rather than neglecting poorly governed nations, our findings stress the need for developing and applying different conservation approaches in different regions [40]. We need to find less costly ways of working in the expensive and well-governed regions (e.g. North America, Australia), perhaps building on volunteer-based conservation programmes, or comprehensive land use planning approaches. Low cost and well-governed regions may be most suitable targets for the traditional external conservation investments. Valuable areas suffering from poor governance (e.g. Sub-Saharan Africa, Papua New Guinea), must not be abandoned, especially, as many of these areas also face the highest predicted loss of suitable habitat for mammals in the future [41]. Africa, in particular, with its high mammal diversity and high predicted future threats in addition to high levels of corruption, presents a specific challenge to the conservation community. Conservation projects might usefully focus on conservation education and engagement of local people, through setting up social-learning institutions [42], with the aim of changing things from within the society. Finally, we want to stress the need for furthering the mechanistic understanding of the effects of governance on conservation effectiveness.
